# The JAK2 pathway is activated in idiopathic pulmonary fibrosis

**DOI:** 10.1186/s12931-018-0728-9

**Published:** 2018-02-06

**Authors:** Javier Milara, Gracia Hernandez, Beatriz Ballester, Anselm Morell, Inés Roger, P. Montero, Juan Escrivá, José M. Lloris, Maria Molina-Molina, Esteban Morcillo, Julio Cortijo

**Affiliations:** 1 Department of Pharmacology, Faculty of Medicine, Jaume I University, Castellon de la Plana, Spain; 2 Pharmacy Unit, University General Hospital Consortium, Valencia, Spain; 3 CIBERES, Health Institute Carlos III, Valencia, Spain; 4 Departmnt of Biotechnology, Valencia Polytechnic University of Valencia, Valencia, Spain; 5 Department of Pharmacology, Faculty of Medicine, University of Valencia, Valencia, Spain; 6 Thoracic Surgery Unit, University and Polytechnic Hospital La Fe, Valencia, Spain; 7 Department of Medicine, Faculty of Medicine, University of Valencia, Valencia, Spain; 8 Unidad Funcional de Intersticio Pulmonar (UFIP), Servicio de Neumología, Hospital Universitario de Bellvitge, IDIBELL, L’Hospitalet de Llobregat, Barcelona, Spain; 9 Health Research Institute INCLIVA, Valencia, Spain; 10 Research and Teaching Unit, University General Hospital Consortium, Valencia, Spain; 11 Unidad de Investigación Clínica, Consorcio Hospital General Universitario, Avenida Tres Cruces s/n, E-46014 Valencia, Spain

**Keywords:** JAK2, STAT3, Idiopathic pulmonary fibrosis, Lung fibroblasts, Alveolar type II epithelial cells

## Abstract

**Background:**

Idiopathic pulmonary fibrosis (IPF) is the most rapidly progressive and fatal fibrotic disorder, with no curative therapies. The signal transducer and activator of transcription 3 (STAT3) protein is activated in lung fibroblasts and alveolar type II cells (ATII), thereby contributing to lung fibrosis in IPF. Although activation of Janus kinase 2 (JAK2) has been implicated in proliferative disorders, its role in IPF is unknown. The aim of this study was to analyze JAK2 activation in IPF, and to determine whether JAK2/STAT3 inhibition is a potential therapeutic strategy for this disease.

**Methods and results:**

JAK2/p-JAK2 and STAT3/pSTAT3 expression was evaluated using quantitative real time-PCR, western blotting, and immunohistochemistry. Compared to human healthy lung tissue (*n* = 10) both proteins were upregulated in the lung tissue of IPF patients (*n* = 12). Stimulating primary ATII and lung fibroblasts with transforming growth factor beta 1 or interleukin (IL)-6/IL-13 activated JAK2 and STAT3, inducing epithelial to mesenchymal and fibroblast to myofibroblast transitions. Dual p-JAK2/p-STAT3 inhibition with JSI-124 or silencing of JAK2 and STAT3 genes suppressed ATII and the fibroblast to myofibroblast transition, with greater effects than the sum of those obtained using JAK2 or STAT3 inhibitors individually. Dual rather than single inhibition was also more effective for inhibiting fibroblast migration, preventing increases in fibroblast senescence and Bcl-2 expression, and ameliorating impaired autophagy. In rats administered JSI-124, a dual inhibitor of p-JAK2/p-STAT3, at a dose of 1 mg/kg/day, bleomycin-induced lung fibrosis was reduced and collagen deposition in the lung was inhibited, as were JAK2 and STAT3 activation and several markers of fibrosis, autophagy, senescence, and anti-apoptosis.

**Conclusions:**

JAK2 and STAT3 are activated in IPF, and their dual inhibition may be an attractive strategy for treating this disease.

**Electronic supplementary material:**

The online version of this article (10.1186/s12931-018-0728-9) contains supplementary material, which is available to authorized users.

## Background

Idiopathic pulmonary fibrosis (IPF) is a chronic, progressive, and fatal form of fibrosing interstitial pneumonia of unknown cause. The typical clinical course includes dyspnea, decreased exercise capacity, dry cough, and death at 2.5–5 years after diagnosis [1]. Although recent studies have provided insights into the pathophysiology of IPF, treatment options for this disease remain limited [1]. A prerequisite for the development of potential therapeutic targets is a better understanding of the pathogenesis of IPF.

IPF is characterized by heterogeneous pulmonary lesions at different stages of evolution, with foci of proliferative fibroblasts and myofibroblasts, abnormal lung epithelial cells, and an overwhelming matrix accumulation in the lung interstitium [2]. The origins of the invasive lung myofibroblasts and their activation are unknown but are probably multiple, including activation of lung resident fibroblasts, recruitment of circulating fibrocyte blood mesenchymal precursors, and mesenchymal transformations of alveolar type II epithelial cells (ATII), endothelial cells, pericytes, and mesothelial cells [3].

Transforming growth factor beta 1 (TGF-β1) is a well-studied, profibrotic growth factor that plays a key role in IPF by driving lung fibroblast activation and promoting mesenchymal transformations of different cell types [2]. However, other important fibrogenic mediators are also elevated in diseased lung tissue and participate in the pathogenesis of IPF [2, 3]. Therefore, an effective treatment for IPF must address its multiple mechanisms.

Tyrosine kinases are a complex, heterogeneous group of cell signal transducers that regulate a wide variety of physiological cell processes including metabolism, growth, differentiation, and apoptosis [4]. Deregulated tyrosine kinase activity can promote the development and progression of neoplastic, cardiovascular, and fibrotic diseases. Currently, the only two drugs approved for the treatment of IPF are pirfenidone and nintedanib [1]. Nintedanib, a multi-tyrosine kinase inhibitor, ameliorates IPF progression and symptoms by blocking the tyrosine kinases coupled to platelet-derived growth factor (PDGF), fibroblast growth factor (FGF), and vascular endothelial growth factor (VEGF) receptors [5, 6]. It is therefore likely that specific tyrosine kinase inhibition can block the signal transduction of multiple key mediators of fibrosis.

Janus kinases (JAKs) are receptor-associated tyrosine kinases with central roles in cytokine and growth factor signaling. Like other receptor-associated tyrosine kinases, cytokine binding induces autophosphorylation and activation of JAK kinases [7]. In turn, JAK kinases recruit and phosphorylate signal transducer and activator of transcription (STAT) proteins. Upon activation, STATs dimerize and translocate to the nucleus, where they activate the transcription of several target genes [7]. Alterations in JAK2 signaling cause profound changes in the cellular response to cytokine stimulation. TGF-β1 signaling induces phosphorylation and activation of JAK2, which then interacts and phosphorylates STAT3 to induce fibrotic responses [8]. In addition, JAK2 can be activated by other profibrotic mediators, including PDGF, VEGF, interleukin (IL)-6, IL-13, angiotensin II (ANGII), serotonin (5-HT), and endothelin (ET-1) [9, 10]. STAT3 phosphorylation has been detected in fibrotic lung tissue from IPF patients and participates in both the fibroblast to myofibroblast transition and lung epithelial cell damage; therefore, it is an attractive therapeutic target in IPF [11–13]. By contrast, the role of JAK2 in IPF has not been studied. Several JAK2 and STAT3 inhibitors are currently being evaluated in clinical trials involving various malignancies and inflammatory diseases. Thus, demonstration of anti-fibrotic effects in experimental models of fibrosis may have direct translational implications. In this study, we established different in vitro and in vivo models relevant to IPF to analyze the participation of JAK2 in IPF and the dependent and independent relationships of this protein with STAT3.

## Methods

See the Online Supplement for more detailed descriptions of these methods.

### Patients

Human lung tissue was obtained from IPF patients who underwent surgery for organ transplantation (*n* = 12). Healthy lung explant control samples were obtained from the organ transplant program of the University General Consortium Hospital of Valencia, Spain. The protocol was approved by the local research and independent ethics committee of the University General Consortium Hospital of Valencia (CEIC21/2013). Informed written consent was obtained from each patient.

### Isolation and culture of human ATII cells and lung fibroblasts and in vitro experimental conditions

Primary ATII cells were obtained from the lung parenchyma of IPF patients as previously outlined [14]. The cells were suspended in Dulbecco’s Modified Eagle’s Medium plus 10% fetal calf serum, 2 mM l-glutamine, 100 U penicillin/mL, and 100 g streptomycin/mL. Primary human lung fibroblasts were obtained from the lung parenchyma of IPF patients with macroscopically fibrotic areas of disease, as previously described [15]. The A549 human alveolar type II cell line and MRC5 normal lung fibroblasts were purchased from the American Type Culture Collection (Rockville, MD, USA) and cultured in supplemented Roswell Park Memorial Institute 1640 medium as outlined [16]. For the in vitro studies, ATII/A549 or primary lung fibroblast/MRC5 were stimulated with recombinant TGF-β1 (5 ng/mL; Sigma Aldrich, St. Louis, MO, USA) or IL-6 (50 ng/mL; Sigma) together with IL-13 (50 ng/mL; Sigma) for the indicated times, replacing the culture medium and stimulus every 24 h. TGF-β1 (5 ng/mL), IL-6 (50 ng/mL), and IL-13 (50 ng/mL) have been shown to induce cell phenotypic changes, including epithelial to mesenchymal transition, at the concentrations used in this study [14, 17, 18]. JSI-124 (Sigma) is a selective JAK2/STAT3 inhibitor that at a concentration of 1 μM suppresses JAK2/STAT3 activation in A549 cells [19]; NSC-33994 (Sigma) is a selective JAK2 inhibitor that at 1 μM completely inhibits JAK2 activity without affecting other tyrosine kinases [20]); 5, 15-DPP (Sigma) is a selective STAT3 inhibitor that at 1 μM completely inhibits STAT3 activity without affecting other STATs [21]. These inhibitors were added 30 min before the stimulus and left in the medium together with the stimulus until the effects were evaluated. Trypan blue staining of the cells was > 95%, which showed that none of the drugs altered viability.

### Western blotting

Changes in the expression levels of proteins in human and rat lung tissues, in ATII/A549, and in lung fibroblast/MRC5 were examined by western blotting. The bands shown on the films were analyzed by densitometry using Image J 1.42q software (available at http://rsb.info.nih.gov/ij/, Bethesda, MD, USA). Target protein levels are expressed as the percentage of the densitometry values of the endogenous control (β-actin).

### Small interfering RNA experiments

Total RNA was isolated from cells/lung tissue using TriPure® isolation reagent (Roche, Indianapolis, IN, USA) as previously described [22]. Small interfering RNAs (siRNAs), including the scrambled siRNA control and JAK2 and STAT3 gene-targeted siRNAs, were designed by Ambion (Huntingdon, Cambridge, UK). A549 and MRC5 cells were transfected with siRNA (50 nM) in serum and antibiotic-free medium as previously reported [23].

### Histological and immunohistochemical studies

Lung histology and immunohistochemistry were conducted as previously reported [24]. Tissue blocks (4 μm thickness) were stained with hematoxylin & eosin to assess fibrotic injury and pulmonary artery remodeling, and with Masson’s trichrome (Sigma-Aldrich, Madrid, Spain) to detect collagen deposition. The severity of lung fibrosis was scored on a scale from 0 (normal lung) to 8 (total fibrotic obliteration of tissue in the examined fields) according to Ashcroft [25]. For immunohistochemical analysis of rat and human lung, the tissues were fixed and embedded in paraffin, cut into sections (4–6 μm), and incubated with JAK2, pJAK2, STAT3, pSTAT3, collagen type I, LC3II, beclin-I, Bcl-2, and p21 antibodies for 24 h at 4 °C. The non-immune IgG isotype control was used as the negative control for all of the samples.

### ELISA

IL-6 and IL-13 cytokines were analyzed in the cell culture supernatants of human ATII and fibroblast using commercially available Quantikine® ELISA kits for human IL-6 (Catalog No. D6050; R&D Systems, Madrid, Spain) and IL-13 (Catalog No. D1300B), and in the bronchoalveolar lavage (BAL) fluid of rats using the ELISA rat IL-6 (Catalog No. KRC0061; Invitrogen™, Madrid, Spain) and IL-13 (Catalog No. KRC0132; Invitrogen™) kits according to the manufacturers’ protocols.

### Wound repair and cell proliferation assay

Wound repair studies were performed in IPF primary human lung fibroblasts as previously outlined [26]. The proliferation of IPF primary human lung fibroblasts was measured in a colorimetric immunoassay based on BrdU incorporation during DNA synthesis, which was performed using a cell proliferation ELISA BrdU kit (Roche, Mannheim, Germany) as previously described [27].

### Micro-computed tomography imaging of intratracheal bleomycin animals and BAL

Animal experiments and handling were performed in accordance with the guidelines of the Committee of Animal Ethics and Well-being of the University of Valencia (Valencia, Spain) as previously outlined [24]. After the rats had been anesthetized with ketamine/medetomidine, a single dose of bleomycin at 3.75 U/kg (dissolved in 200 μL saline) was administered intratracheally via the endotracheal route [28]. Sham-treated rats received the identical volume of intratracheal saline instead of bleomycin. This procedure defined day 1 of the experiment. The dose of intraperitoneally administered JSI-124 (1 mg/kg/day) was selected based on the results of previous in vivo animal studies [29]. The inhibitor was administered from day 14 to day 28 as a therapeutic protocol. BAL fluid was processed as previously outlined, and the contents of inflammatory cells, protein, and IL-6/IL-13 were measured [30]. Micro-computed tomography (micro-CT) analyses were performed as previously reported [31]. Densitometric analysis of the extension of fibrosis was performed using the micro-CT images, with the density expressed as Hounsfield units.

### Statistical analysis

The results were statistically analyzed using non-parametric tests (human tissue studies) and expressed as medians and interquartile ranges. In comparisons of two groups, between-group differences were analyzed using the Mann–Whitney test. Parametric tests were used to analyze the data obtained in animal and cellular in vitro mechanistic experiments; the results are expressed as the mean ± SEM of *n* experiments. Two-group comparisons were analyzed using a two-tailed Student’s paired *t*-test for dependent samples, and an unpaired *t*-test for independent samples. Multiple comparisons were made using a one-way or two-way analysis of variance followed by a Bonferroni *post-hoc* test. A *P* value < 0.05 was considered statistically significant.

## Results

### JAK2 and STAT3 are increased and activated in the lungs of IPF patients

**Fig. 1:**
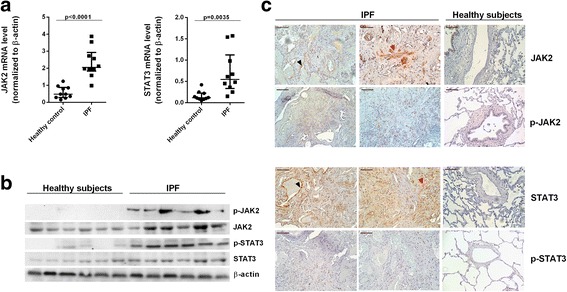
Expression and localization of JAK2, STAT3, and their phosphorylated forms in lung tissue from patients with IPF. JAK2 and STAT3 gene expression and JAK2/p-JAK2 and STAT3/p-STAT3 protein expression were analyzed in lung tissue from healthy controls (*n* = 10) and IPF patients (*n* = 12) by (**a**) qPCR, (**b**) western blotting, and (**c**) immunohistochemistry. Scale bar: 100 μm. Black arrows indicate hyperplastic alveolar type II cells, and red arrows indicate fibroblasts. Expression was calculated as the ratio compared to β-actin expression. The data are presented as a scatter dot plot showing the median and interquartile range. Exact values for *P* were obtained using the Mann–Whitney test

Both control and IPF patients were prospectively recruited from the Thoracic Surgery and Pathology Services of the University General Consortium Hospital and University and Polytechnic Hospital La Fe (Valencia, Spain) between 2013 and 2016 at the initial diagnostic work-up. The clinical data of the patients are shown in Table 1. In homogenized lung tissue, JAK2 and STAT3 mRNA transcript levels were both higher in that of IPF patients than in that of controls (*p* < 0.001 and *p* = 0.0035, respectively) (Fig. 1a), as were JAK2 and STAT3 protein expression. In addition, whereas the active phosphorylated forms of JAK2 and STAT3 were upregulated in IPF lung tissue, the levels were almost undetectable in control lung tissue (Fig. 1b). Immunohistochemistry of control lung sections showed weak JAK2 and STAT3 expression that was localized mainly to bronchial epithelium and alveolar macrophages (Fig. 1c). By contrast, in IPF lung tissue, expression of both markers was elevated in hyperplastic alveolar cells (Fig. 1c, red arrows) and fibroblasts (Fig. 1c, black arrows). The localization of phosphorylated JAK2 and STAT3 in the nuclei of cells in fibrotic areas of the lung implies that these proteins function as transcription factors.

**Table 1:** Clinical features

	Control donor subjects (*n* = 10)	IPF patients (*n* = 12)
Age (yr)	57 [38–65]	66 [58–75]
Sex (M/F)	6/4	9/3
Smoking		
*Never smoked/Smokers*	3/7	1/11
*Pack-year*	25 [0–28]	26.3 [11–34]
FEV1, pred	ND	72.2 [56–96]
FVC, % pred	ND	62.2 [55–68]
TLC, % pred	ND	73.5 [45–89]
DLco, % pred	ND	42.1 [31–51]
Ground glass %	0	25 [20–39]
Honeycombing %	0	35 [25–40]
NAC (y/n)	0/10	8/4
Pirfenidone (y/n)	0/10	4/8

*% pred* % predicted, *DLco* diffusion capacity of the lung for carbon monoxide, *FEV1* forced expiratory volume in 1 s, *FVC* forced vital capacity, *ND* not determined, *Pack-year* 1 year of smoking 20 cigarettes per day, *TLC* total lung capacity, *Ground glass %* % of pulmonary parenchyma with ground glass on a computed tomography (CT) image, *Honeycombing %* % of pulmonary parenchyma with honeycombing on a CT image; N-acetyl-l-cysteine (NAC). Data are medians [interquartile range]

### Phosphorylation of JAK2 and STAT3 induces mesenchymal transition in ATII and transition of fibroblasts to myofibroblasts in the lung

**Fig. 2:**
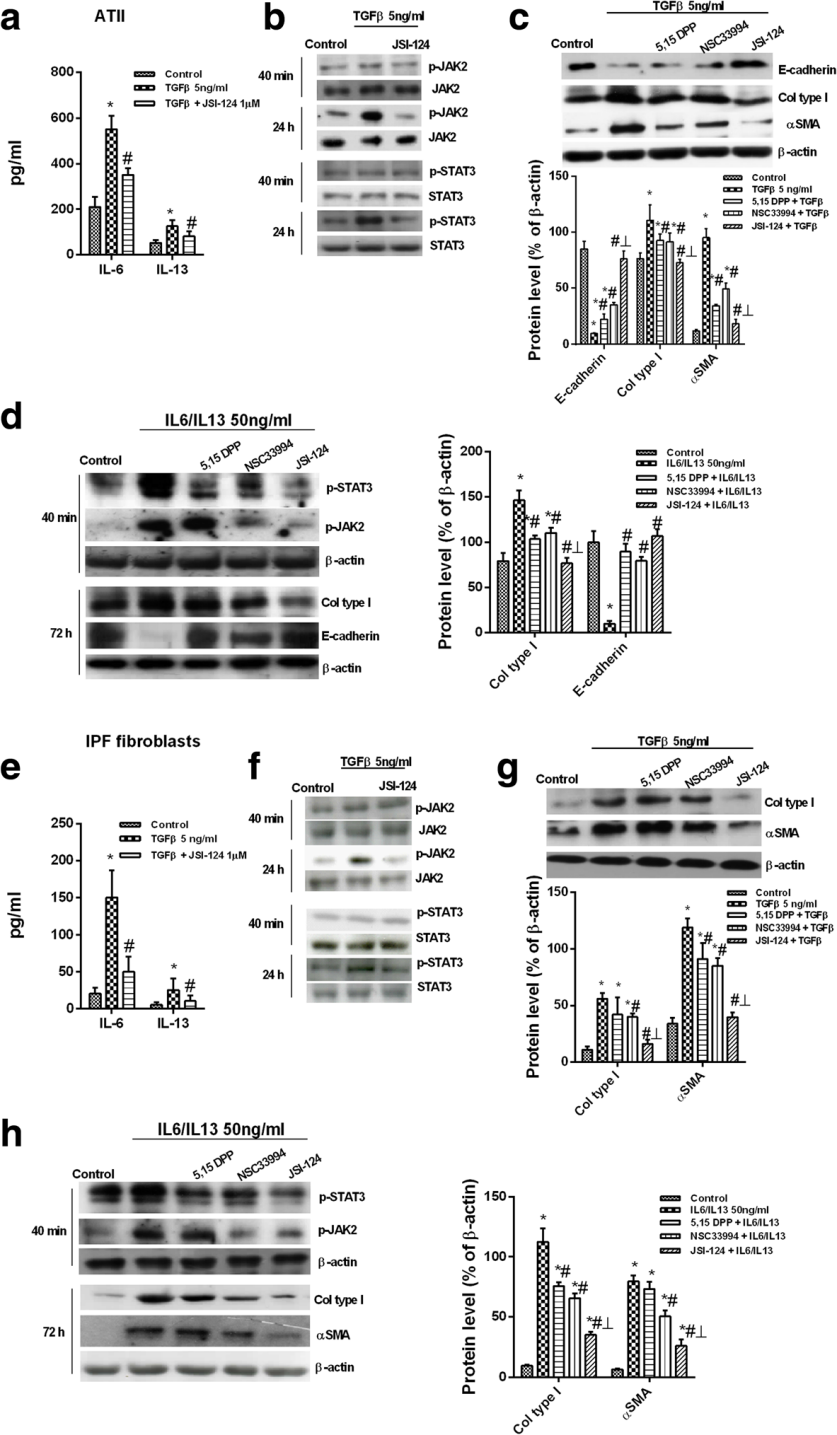
Effects of JAK2 and STAT3 on ATII to mesenchymal and fibroblast to myofibroblast transitions. Primary ATII and lung fibroblasts were isolated from the lungs of IPF patients. **a** The cells were incubated with the dual p-JAK2/p-STAT3 inhibitor JSI-124 for 30 min followed by TGF-β1 stimulation for an additional 24 h. IL-6 and IL-13 levels in cell supernatants were measured using ELISA. **b** Ratios of JAK2/p-JAK2 and STAT3/p-STAT3 were determined by western blotting in ATII cells stimulated for 40 min or 24 h with TGF-β1 in the presence or absence of JSI-124. **c**, **d** ATII cells were pre-incubated for 30 min with 1 μM of the p-STAT3 inhibitor 5,15 DPP, the p-JAK2 inhibitor NSC33994, or the dual p-JAK2/p-STAT3 inhibitor JSI-124, and then stimulated for 72 h with TGF-β1 (**c**) or IL-6/IL-13 (**d**). **e** Levels of IL-6 and IL-13 in primary fibroblasts. **f** JAK2/p-JAK2 and STAT3/p-STAT3 protein expression in human lung fibroblasts. **g**, **h** Primary lung fibroblasts pre-incubated for 30 min with 1 μM of the p-STAT3 inhibitor 5,15 DPP, the p-JAK2 inhibitor NSC33994, or the dual p-JAK2/p-STAT3 inhibitor JSI-124 and stimulated for 72 h with TGF-β1 (**g**) or IL-6/IL-13 (**h**). Representative western blots are shown. The results are expressed as the mean (SEM) of *n* = 4 (cells from four IPF patients) experiments per condition. Two-way ANOVA followed by *post-hoc* Bonferroni tests. *P < 0.05 vs. the solvent controls; ^#^*P* < 0.05 vs. TGF-β1 or IL-6/IL-13; ┴ *P* < 0.05 vs. 5,15 DPP or NSC33994

In IPF tissue, TGF-β1 significantly increased IL-6 and IL-13 release from ATII inhibited by JSI-124 (Fig. 2a), but after 40 min of stimulation, neither JAK2 nor STAT3 was phosphorylated. However, after 24 h stimulation (Fig. 2b), TGF-β1 enhanced p-JAK2 and p-STAT3 levels. It also promoted ATII to mesenchymal transition, increasing the mesenchymal markers αSMA and collagen type I and downregulating the epithelial marker E-cadherin (Fig. 2c). These changes were attenuated by specific p-STAT3 and p-JAK2 inhibitors 5,15 DPP and NSC33994, and suppressed by the dual p-JAK2/p-STAT3 inhibitor JSI-124 (Fig. 2c). Stimulation of ATII cells with a combination of IL-6/IL-13 increased p-JAK2 and p-STAT3 expression (Fig. 2d). The phosphorylation of both proteins was inhibited by JSI-124 and NSC33994. However, the p-STAT3 inhibitor 5, 15 DPP inhibited only STAT3, not JAK2 phosphorylation (Fig. 2d). The IL-6/IL-13 combination also increased expression of mesenchymal markers in ATII cells, including collagen type I protein and mRNA as well as αSMA, Snail, and Slug mRNA, and decreased expression of the epithelial marker E-cadherin (Fig. 2d and Additional file 1: Figure S1). The dual p-JAK2/p-STAT3 inhibitor JSI-124 suppressed ATII to mesenchymal transition whereas the inhibitory effects of NSC33994 and 5, 15 DPP were weaker (Fig. 2d). Similar results were obtained in primary lung fibroblasts from IPF patients. TGF-β1 significantly increased IL-6 and IL-13 release from lung fibroblasts, and after 24 h stimulation phosphorylated JAK2 and STAT3 (Fig. 2e and f). JSI-124 inhibited TGF-β1-induced IL-6 and IL-13 release from lung fibroblasts as well as JAK2/STAT3 phosphorylation. TGF-β1 promoted fibroblast to myofibroblast transition, which was partially inhibited by NSC33994 and 5, 15 DPP and completely suppressed by JSI-124 (Fig. 2g). Combination of IL-6 and IL-13 promoted fibroblast to myofibroblast transition, increasing expression of collagen type I, αSMA, Snail, and Slug. The latter effect was suppressed by JSI-124, and to a lesser extent by NSC33994 and 5, 15 DPP (Fig. 2h and Additional file 1: Figure S1).

**Fig. 3:**
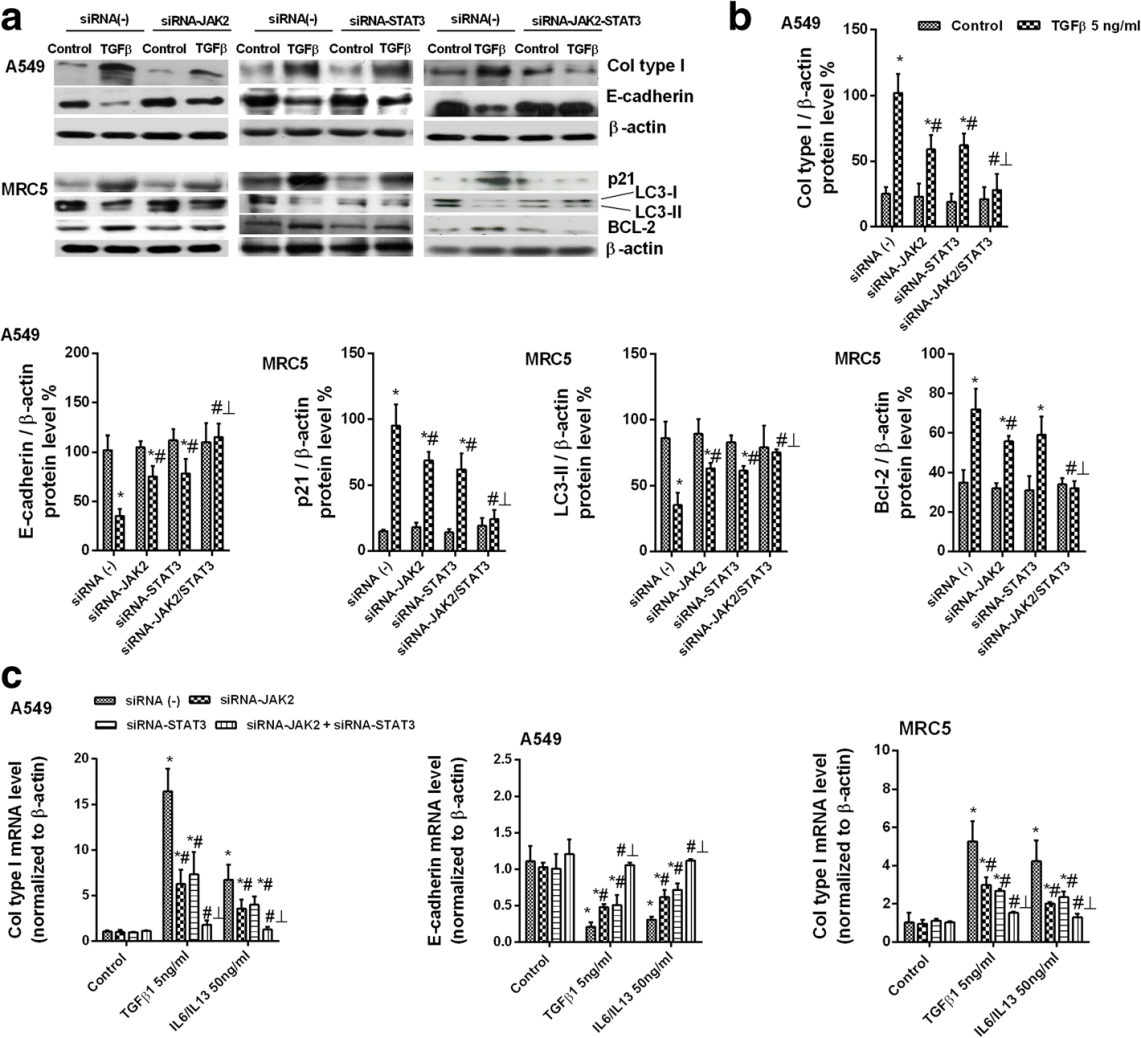
Dual JAK2/STAT3 gene silencing suppressed ATII to mesenchymal and fibroblast to myofibroblast transitions. ATII A549 and human lung fibroblast MRC5 cell lines were transfected with control siRNA(−), siRNA-JAK2, siRNA-STAT3, or both siRNA-JAK2/STAT3 and stimulated for 72 h with TG-Fβ1 or IL-6/IL-13 at a concentration of 5 ng/mL. **a** Total protein and RNA from cell lysates were analyzed by (**a**, **b**) western blot and (**c**) qPCR. Mesenchymal collagen type I and epithelial E-cadherin markers were measured using (**a**) western blot and (**c**) qPCR. Senescence p21, autophagy LC3I/II, and anti-apoptotic BCL-2 markers were (**a**) measured using western blot and (**b**) quantified by densitometry. Data are expressed as the ratio to β-actin protein and mRNA levels. The results are expressed as the mean (SEM) of *n* = 4 independent experiments per condition. One-way ANOVA followed by *post-hoc* Bonferroni tests. *P < 0.05 vs. the solvent controls; ^#^*P* < 0.05 vs. TGF-β1 or IL-6/IL-13; ┴ *P* < 0.05 vs. siRNA-JAK2 or siRNA-STAT3

**Fig. 4:**
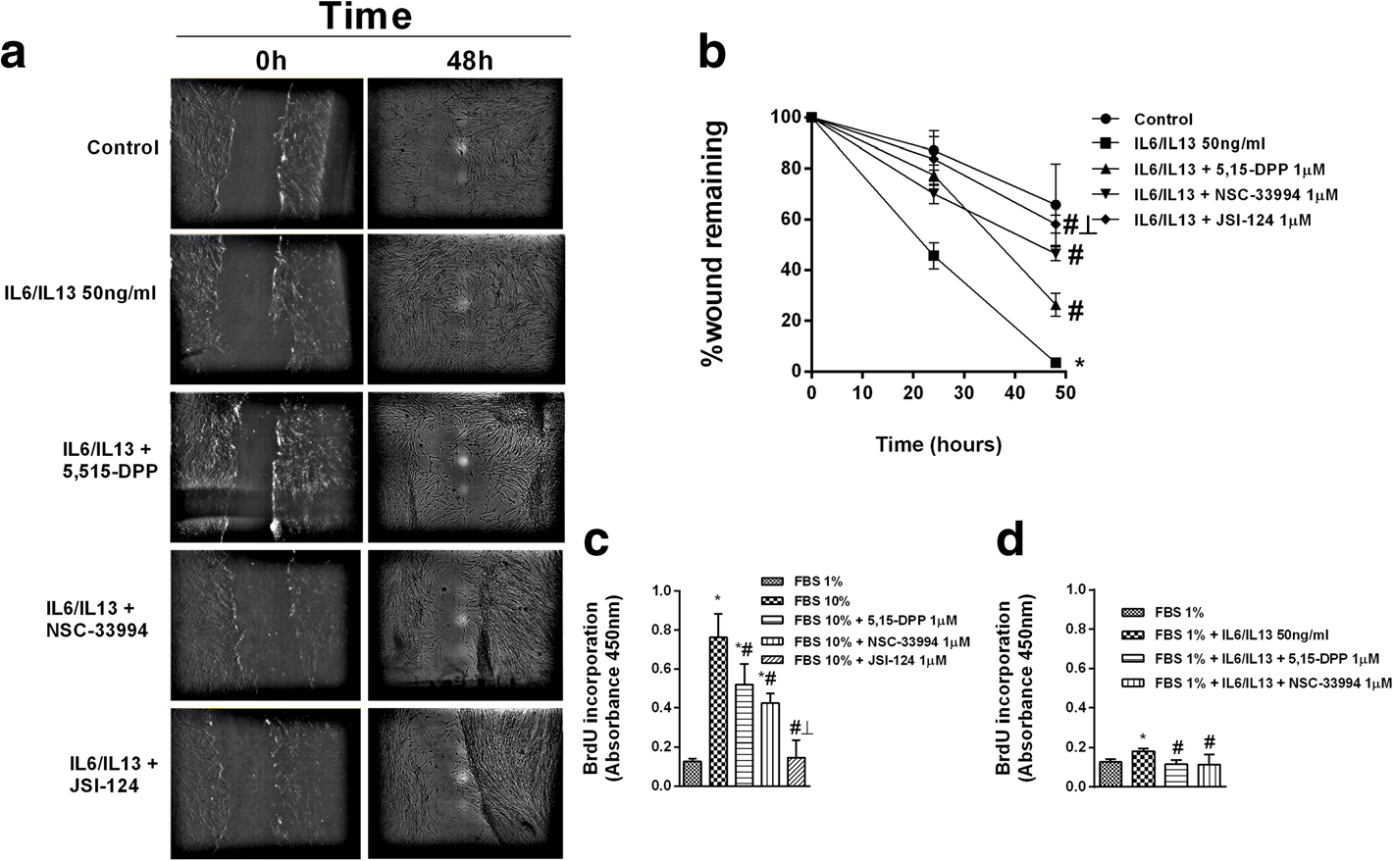
Fibroblast migration and proliferation in IPF were dependent on JAK2 and STAT3 activation. **a**, **b** Primary human fibroblasts from IPF patients were pre-treated for 30 min with 1 μM of the p-STAT3 inhibitor 5,15 DPP, the p-JAK2 inhibitor NSC33994, or the dual p-JAK2/p-STAT3 inhibitor JSI-124 and then cell migration was assessed. A scrape-wound was created by using a sterile p200 pipette tip to make a perpendicular linear scratch in the culture. After the cells had been washed, culture medium with or without pharmacologic modulators and IL-6/IL-13 was added. The size of the wound remaining was analyzed at the indicated times and expressed as a percentage of the initial wound area. **c** Fibroblast proliferation during 48 h was evaluated by the BrdU assay. Different JAK2 and STAT3 inhibitors were added 30 min before 10% fetal bovine serum (FBS) or (**c**) 50 ng IL-6/IL-13/mL (**d**) was added as the stimulus. Values are expressed as relative absorbance (450 nm) units. The results are expressed as the mean (SEM) of *n* = 4 (cells from four IPF patients) experiments per condition. Two-way ANOVA followed by *post-hoc* Bonferroni tests. *P < 0.05 vs. the solvent controls; ^#^*P* < 0.05 vs. IL-6/IL-13 or 10% FBS; ┴ *P* < 0.05 vs. 5,15 DPP or NSC33994

In the A549 alveolar type II cell line, TGF-β1 increased collagen type I and reduced E-cadherin protein expression, with both responses partially inhibited in cells selectively transfected with siRNA-JAK2 or siRNA-STAT3, and completely suppressed by dual siRNA-JAK2/STAT3 transfection (Fig. 3a and b). Similar results were obtained using TGF-β1 or IL-6/IL-13 as an enhancer of epithelial to mesenchymal transition, as both increased collagen type I and E-cadherin gene mRNA expression (Fig. 3c). TGF-β1 promoted increases in the senescence of p21 fibroblasts and expression of Bcl-2 anti-apoptotic markers but also decreases in LC3I/II autophagy markers in A549 alveolar type II cells. These responses were partially inhibited by incubation of the cells with siRNA-JAK2 or siRNA-STAT3, and completely suppressed by dual si-RNA-JAK2/STAT3 transfection (Fig. 3a and b). Both TGF-β1 and IL-6/IL-13 increased collagen type I gene expression in MRC5 cells, whereas siRNA-JAK2 and siRNA-STAT3 partially inhibited this response and dual siRNA-JAK2/STAT3 transfection completely inhibited this response (Fig. 3c). IPF fibroblasts stimulated with IL-6/IL-13 accelerated wound repair in vitro, indicative of the increased migration capacity of these cells. JAK2 inhibition, and to a lesser extent STAT3 inhibition, reduced fibroblast migration, which was almost completely suppressed to control levels after inhibition of JAK2/STAT3 by JSI-124 (Fig. 4a and b). JSI-124 was more effective than single inhibitors of JAK2 or STAT3 in blocking cell proliferation in cultures containing fetal bovine serum (Fig. 4c). IL6/IL13 showed only weak effects on fibroblast proliferation suppressed by JAK2 and STAT3 inhibitors (Fig. 4d).

### Effects of JAK2/STAT3 dual inhibition on bleomycin-induced lung fibrosis

**Fig. 5:**
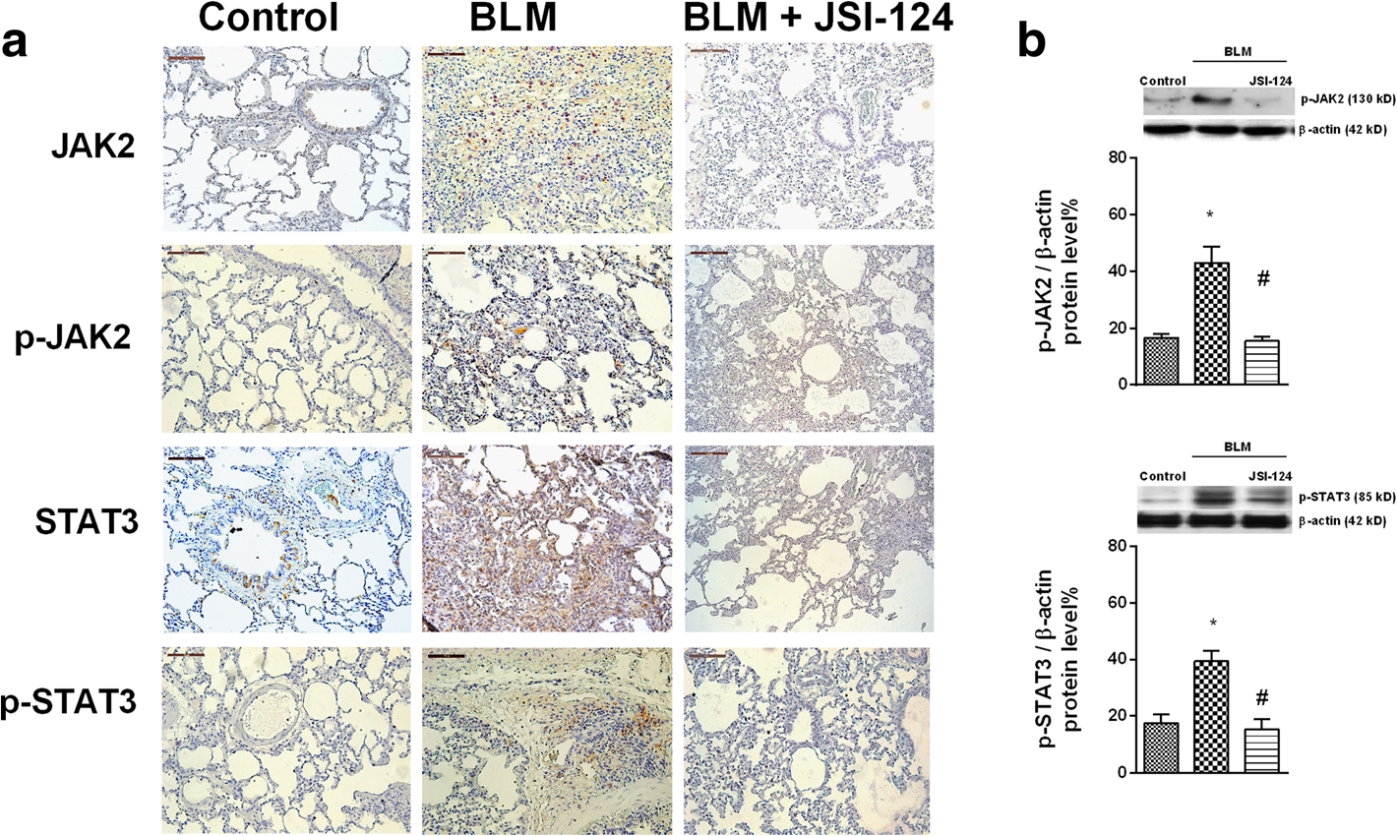
Bleomycin induced lung fibrosis and JAK2 and STAT3 activation. Wistar rats were administered a single intratracheal dose of bleomycin (BLM; 3.75 U/kg) on day 1. JSI-124 (1 mg/kg/day) or vehicle was administered intraperitoneally from day 14 until the analysis at day 28 (*n =* 10 per group). Lung tissue from the vehicle control, BLM, and BLM + JSI-124 groups was immunostained for JAK2, p-JAK2, STAT3, and p-STAT3 (brown) and counterstained with hematoxylin. Representative (**a**) immunohistochemistry and (**b**) western blot images are shown. Scale bar = 100 μm. The IgG isotype control was negative (data not shown). Data are expressed as the ratio to β-actin for protein levels. The results are expressed as the mean (SEM), *n =* 10. One-way ANOVA followed by *post-hoc* Bonferroni tests. *P < 0.05 vs. controls; ^#^*P* < 0.05 vs. BLM

**Fig. 6:**
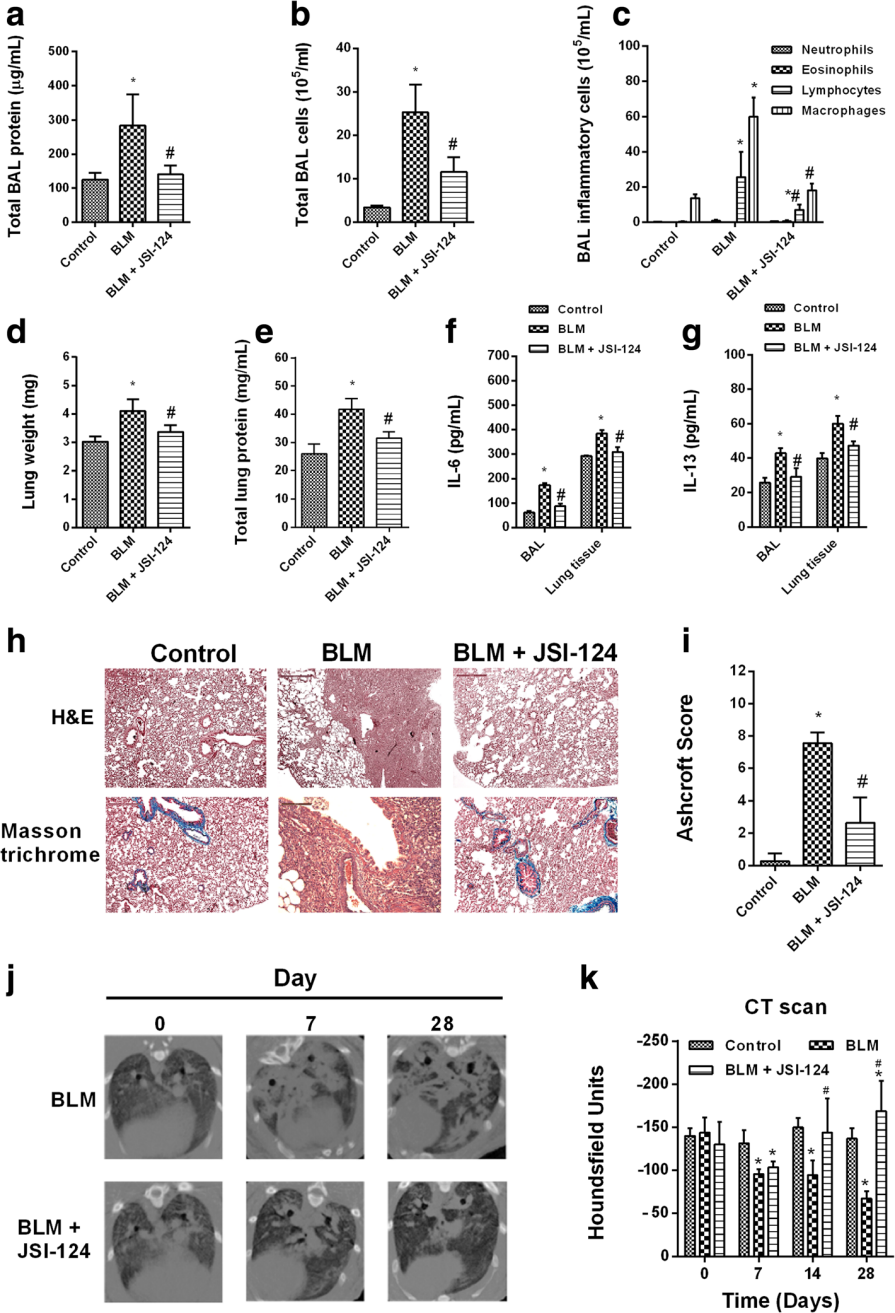
JSI-124 inhibited bleomycin-induced bronchoalveolar inflammatory cell extravasation and lung fibrosis. Wistar rats were administered a single intratracheal dose of bleomycin (BLM; 3.75 U/kg) on day 1. JSI-124 (1 mg/kg/day) or vehicle was administered intraperitoneally from day 14 until the analysis at day 28 (*n =* 10 per group). Total bronchoalveolar lavage (**a**) protein, (**b**, **c**) inflammatory cells, (**d**) lung weight, (**e**) protein, and (**f**, **g**) IL-6 and IL-13 content were measured at day 28. **h** Masson’s trichrome (upper panels, scale bar: 100 μm) of control, BLM, and BLM + JSI-124 tissue. **i** Fibrosis Ashcroft scores were assessed as described in the Methods. **j** Micro-CT images were acquired on day 28 and (**k**) quantified as Hounsfield units. The results are expressed as the mean ± SEM, *n =* 10. Statistical significance was assessed using a *t*-test or one-way ANOVA followed by a Bonferroni *post-hoc* test. *P < 0.05 vs. control, ^#^*P* < 0.05 vs. BLM

**Fig. 7:**
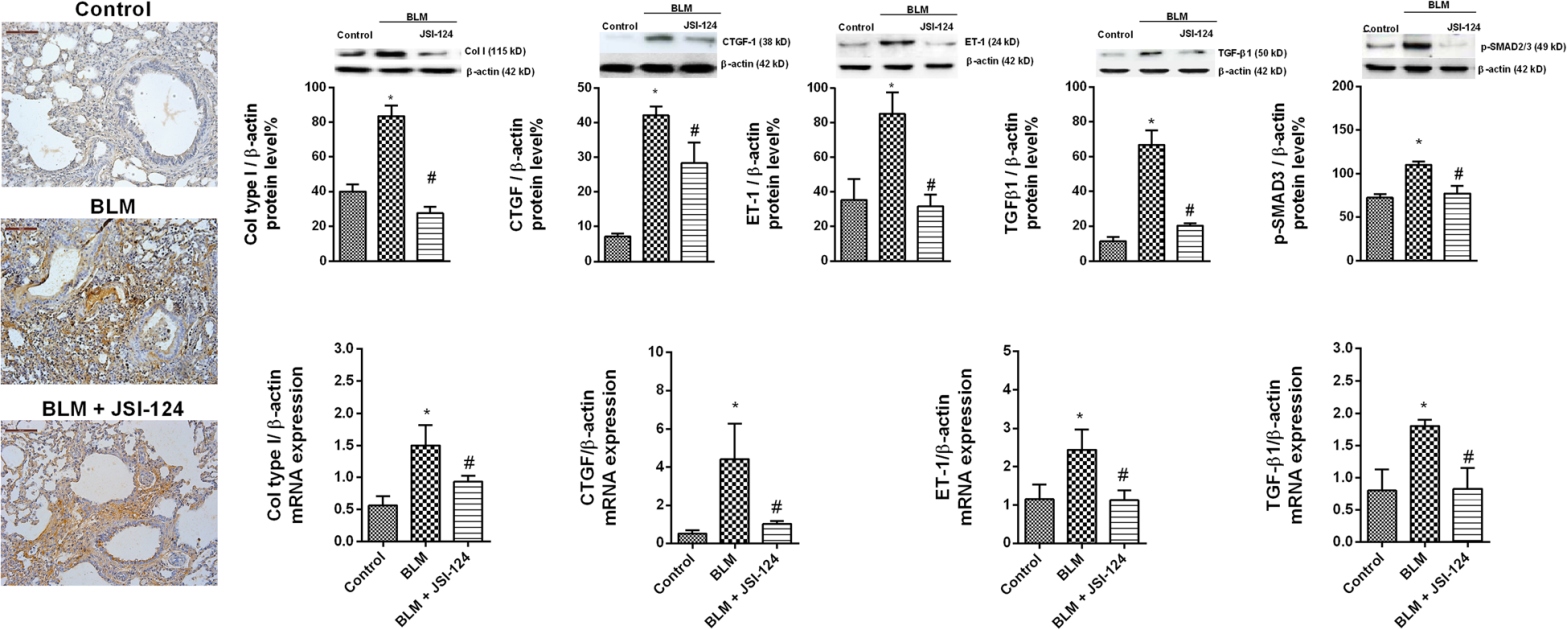
JSI-124 inhibited the bleomycin-induced expression of molecular markers of lung fibrosis. Wistar rats were administered a single intratracheal dose of bleomycin (BLM; 3.75 U/kg) on day 1. JSI-124 (1 mg/kg/day) or vehicle was administered intraperitoneally from day 14 until the analysis at day 28 (*n =* 10 per group). Representative images of lung tissue from control vehicle, BLM, and BLM + JSI-124 groups subjected to immunohistochemistry for collagen type I, western blotting, and qPCR. Scale bar = 100 μm. The IgG isotype control was negative (data not shown). The data are expressed as the ratio compared to β-actin for protein and qPCR levels. The results are expressed as the mean (SEM), *n =* 10. One-way ANOVA followed by *post-hoc* Bonferroni tests. *P < 0.05 vs. controls; #*P* < 0.05 vs. BLM

Phosphorylation of JAK2 and STAT3 in rat lung tissue increased after 28 days of intratracheal bleomycin instillation compared to control animals (Fig. 5). Phosphorylated forms of JAK2 and STAT3 were not detected in control animals, but were increased in the lung tissue of bleomycin-treated rats and located in the nuclei of fibrotic cells. In control animals, non-phosphorylated JAK2 and STAT3 were localized in bronchial epithelial cells, whereas their expression was enhanced in the fibrotic cells of bleomycin-treated rats. Daily intraperitoneal administration of JSI-124 (1 mg/kg) from day 14 to 28 reduced JAK2 and STAT3 phosphorylation (Fig. 5) as well as the increased protein levels and cell numbers in BAL, lung mass and protein, and expression of IL-6 and IL-13 secondary to lung fibrosis (Fig. 6a–g). Bleomycin induced a fibrotic response in the lung that was characterized by increased deposition of collagen, as seen on Masson’s trichrome-stained sections, and a dense fibrotic content, evident on micro-CT images (Fig. 6h–k). Expression of several markers of fibrosis, including collagen type I, connecting tissue growth factor (CTGF), ET-1, TGF-β1, and p-SMAD3, was also increased in bleomycin-treated rats and reduced by JSI-124 treatment (Fig. 7). The latter alleviated the histologically observed, multifocal fibrotic lesions, resulting in smaller, less-organized foci, less septal enlargement, and accordingly a diminished Ashcroft fibrosis score (Fig. 6i). Levels of the autophagy marker LC3I/II and of beclin-1 decreased, and senescence p21 and anti-apoptotic Bcl-2 increased in the lungs of bleomycin-treated rats (Additional file 1: Figure S2). JSI-124 increased autophagy while decreasing the levels of p21 senescence and Bcl-2 anti-apoptotic markers (Additional file 1: Figure S2).

## Discussion

This study examined the role of JAK2 and STAT3 in IPF. Increased STAT3 phosphorylation, which modulates ATII and lung fibroblast plasticity [11, 12], is characteristically detected in lung biopsy tissue from IPF patients, but there is no evidence implying the involvement of JAK2 in IPF. Our results show overexpression of JAK2 and its phosphorylated form in the fibrotic lungs of IPF patients, thus paralleling previous STAT3 findings. JAK2 and STAT3 activation contributed to cell transformations typical of IPF, including the ATII to mesenchymal and fibroblast to myofibroblast transitions and fibroblast proliferation and migration. Dual JAK2/STAT3 inhibition was more effective for inhibiting both these cellular transitions and lung fibrosis than the individual inhibition of JAK2 or STAT3, which implies synergistic and independent roles of these proteins in IPF.

In this study, non-phosphorylated forms of STAT3 were overexpressed in lung tissue from IPF patients and localized in the cytoplasm of fibroblasts from fibrotic areas and in hyperplastic ATII cells, as previously reported [12]. The nuclear localization of p-STAT3 in fibrotic areas of lungs from IPF patients is consistent with its role as a transcription factor modulating the expression of the genes that cause fibrosis [11]. Similar to STAT3, non-phosphorylated JAK2 was overexpressed in the fibrotic lungs of IPF patients and localized both in fibroblasts from fibrotic areas and in ATII hyperplastic cells, which implies a dominant role for these cells in IPF. Surprisingly, the active phosphorylated form of JAK2 was not detected in control healthy lungs, but was also overexpressed and localized in the nuclei of cells from the fibrotic areas of the lungs of IPF patients and bleomycin-treated animals. These are novel observations, as JAK2 and its phosphorylated form are typically located in the cytoplasm. JAK2 is phosphorylated in response to cell stimulation by different cytokines or growth factors, including TGF-β1, which leads to the translocation of STAT3 to the nucleus, where it activates genes associated with fibrosis [8]. The similar nuclear localization of p-JAK2 in fibrotic areas implies that it is a non-canonical transcription factor, with a pathway independent of the canonical STAT3 pathway. Previous reports of the nuclear localization of p-JAK2 support our findings [32, 33], while emerging evidence indicates that nuclear pJAK2 plays important roles in physiological and pathological conditions characterized by heightened cellular growth. Therefore, p-JAK2 activates not only STAT3 but also different intracellular receptors and forms multiprotein complexes [33]. However the exact role of nuclear p-JAK2 in IPF is beyond the scope of the present work. Consistent with an independent role of p-JAK2 in lung fibrosis, we observed that p-JAK2 inhibition in ATII and lung fibroblasts from IPF patients partially reduced the mesenchymal-myofibroblast transformation induced by TGF-β1 and IL-6/IL-13. The degree of inhibition was similar to that of p-STAT3 inhibition. Moreover, the inhibition of p-JAK2/STAT3, whether by JSI-124 or by gene silencing, was synergistic in its inhibitory effects on cell transformation.

Overexpression of p-JAK2 has also been reported in the cytoplasm of skin fibroblasts from systemic sclerosis (SSc) patients [8]. In SSc, TGF-β1 independently activates JAK2 and STAT3 via SMAD3, and pharmacologic or genetic inactivation of JAK2 in skin reduces the profibrotic effects of TGF-β1 [8]. However, recent evidence indicates that in fibroblasts from IPF patients, TGF-β1 activates STAT3 via a SMAD2/3-dependent mechanism, independent of JAK2 [12]. A physical interaction between STAT3 and the TGF-β receptor I and between STAT3 and SMAD3 in different cell lines has been suggested [34]. Alternatively, TGF-β1 may activate STAT3 indirectly by inducing IL-6, IL-13, or other activators [35]. In this study, TGF-β1 was shown to increase IL-6 and IL-13 secretion in IPF ATII and fibroblasts after 24 h, to phosphorylate JAK2 and STAT3, which implies either slow or indirect activation of these proteins. In addition to TGF-β1, other important fibrogenic mediators with increased expression in IPF lung tissue were CTGF, PDGF, FGF-2, ET-1, ANGII, and cytokines such as IL-13, IL-6, and chemokine ligand 2, all of which have been implicated in the pathogenesis of this disease [2, 3]. Of note, most mediators activate JAK2 or STAT3 [9, 10]. In this study, bleomycin-induced pulmonary fibrosis was characterized by increases in CTGF, TGFβ1, ET-1, IL-6, and IL-13, as profibrotic mediators. Thus, inhibition of downstream JAK2 and STAT3 signaling by JSI-124 reduced both pulmonary fibrosis and expression of these mediators in the lung. Accordingly, inhibition of JAK2 and STAT3, and thus of several of the cellular pathways implicated in IPF, may be a strategy for treating this complex disease.

Senescence and impaired autophagy are hallmarks of fibroblasts isolated from IPF patients. Autophagy, which helps to maintain the balanced synthesis, degradation, and recycling of organelles and proteins to meet metabolic demands, plays an important regulatory role in cellular senescence and differentiation. Impaired autophagy and increased senescence promote myofibroblast formation in IPF and thus are attractive targets in its treatment. In this work, TGFβ1 increased p21 senescence and Bcl-1 anti-apoptotic markers while decreasing the autophagy marker LC3-I/II in human fibroblasts. Similar results were observed in fibrotic lung tissue from bleomycin-treated rats. As in their cellular transformations, JAK2 and STAT3 exhibited independent effects on autophagy and senescence, with dual JAK2 and STAT3 inhibition leading to greater reductions in cell senescence and higher levels of autophagy than achieved by inhibiting either protein alone. Previous reports have demonstrated a role for STAT3 in fibroblast senescence [36], consistent with the relevance of the JAK2/STAT3 pathway in IPF. However, the individual mechanisms by which JAK2 and STAT3 cause pulmonary fibrosis are currently unknown, and together with the mechanism underlying the synergistic effects of dual inhibition, remain to be determined in future research.

## Conclusion

Levels of activated JAK2 and STAT3 are elevated in lung fibroblasts and ATII cells from IPF patients. These two proteins participate in lung fibrosis by dependent and independent mechanisms that may be targetable in the treatment of IPF. Because JAK2 and STAT3 inhibitors are currently being evaluated in clinical trials for malignancies and inflammatory diseases, the results provided in this study may have direct translational implications.

## Additional file

Additional file 1: Supplementary data. (DOCX 1218 kb)

## Abbreviations

5HT: Serotonin
ANGII: Angiotensin II
ATII: Alveolar type II cells
CTGF: Connecting tissue growth factor
ET-1: Endothelin
FGF: Fibroblast growth factor
IPF: Idiopathic pulmonary fibrosis
JAK2: Janus kinase 2
PDGF: Platelet-derived growth factor
siRNA: Small interfering RNA
SSc: Systemic sclerosis
STAT: Signal transducer and activator of transcription
TGFβ1: Transforming growth factor beta 1
VEGF: Vascular endothelial growth factor
