# Supplementary material for: The JAK2 pathway is activated in idiopathic pulmonary fibrosis
Source: Respir Res. 2018 Feb 6;19:24. doi: 10.1186/s12931-018-0728-9 (PMC5801676; doi:10.1186/s12931-018-0728-9)
Supplement: Additional file 1: — Supplementary data. (DOCX 1218 kb) [file 12931_2018_728_MOESM1_ESM.docx]

**Supplemetary Methods and Figures**

**Title**

**JAK2 PATHWAY IS ACTIVATED IN IDIOPATHIC PULMONARY FIBROSIS**

**Authors**

Javier Milara,^1,2,3,*^ Gracia Hernandez,^4*^ Beatriz Ballester,^5,^ Anselm Morell,^5^ Inés Roger,^3^ Juan Escrivá,^6^ José M Lloris,^7^ Esteban Morcillo,^3,4,8^ Julio Cortijo^3,4,9^

**Affiliations**

^1^Department of pharmacology, faculty of medicine, Jaume I University

^2^Pharmacy Unit, University General Hospital Consortium, Valencia, Spain

^3^CIBERES, Health Institute Carlos III, Valencia, Spain

^4^Departmnt of biotechnology, Valencia Polytechnic University of Valencia, Spain

^5^Department of Pharmacology, Faculty of Medicine, University of Valencia, Spain

^6^Thoracic surgery unit, University and Polytechnic Hospital La Fe, Valencia, Spain.

^7^Medicine department, Faculty of Medicine, University of Valencia

^8^Health Research Institute INCLIVA, Valencia, Spain

^9^Research and teaching Unit, University General Hospital Consortium, Valencia, Spain

*Both authors contributed equally to this work

**Corresponding author:**Javier Milara, PhD; Unidad de Investigación Clínica, Consorcio Hospital General Universitario. Avenida tres cruces s/n; E-46014 Valencia, Spain; Phone: +34 620231549, Fax: +34961972145: E-mail: xmilara@hotmail.com

**MATERIALS AND METHODS**

**Patients**

# Human lung tissue was obtained from 2 types of patients (Thoracic Surgery and Pathology Services of the University General Consortium Hospital (CHGUV) and University and Polytechnic Hospital La Fe, Spain): A) Patients with IPF who were underwent surgery for organ transplantation program (n=12). B) Lung explant control samples were obtained from organ transplant program from CHGUV, in donors with normal lung function that was not used for transplant purposes (represents lung tissue without IPF and were used as controls), without any lung disease (n=10). IPF was diagnosed according to the American Thoracic Society/European Respiratory Society (ATS/ERS) consensus criteria ^[1](#_ENREF_1)^. All pulmonary function tests were performed within 3 months before surgery. After selection based on diagnosis criteria, all lung tissue samples used for the study were checked histologically by using the following exclusion criteria: (1) presence of tumor, (2) respiratory tract infection.

The lungs taken from controls showed normal architecture with few intra-alveolar macrophages and edema. The protocol was approved by the local research and independent ethics committee of the University General Consortium Hospital of Valencia (CEIC21/2013). Informed written consent was obtained from each participant.

**Isolation and culture of human alveolar type II cells and lung fibroblast and *in vitro* experimental conditions**

Primary alveolar type II (ATII) cells were obtained from lung parenchyma of IPF patients as previously outlined ^[2](#_ENREF_2)^. Lung parenchyma tissue was cut in approximately 1 mm thick sections and lavaged with saline. The lung sections were digested with 0.25% trypsin (T8003; Sigma, St. Louis, MO) dissolved in saline (100 ml) and suspended in 0.9% NaCl at 37ºC for 30 minutes. After digestion, the lung sections were treated with DNase dissolved in saline (7,500 U/100 ml), and filtered through nylon meshes ranging in pore size from 150 to 30 mm. The resulting cell suspension was centrifuged (250 x g, 20 min at 10ºC) through a sterile Percoll gradient and the alveolar type II cell–rich band was removed. A second DNase treatment (2,000 U/100 ml) was administered and the cells were recovered as a pellet by centrifugation at 250 x g for 20 minutes. These cells were resuspended in 5 ml of DCCM-1 (Biological Industries, Kibbutz Beit Haemek, Israel) supplemented with a 2% (wt/vol) L-glutamine and subjected to differential attachment on a plastic Petri dish. No adherent alveolar type II cells were collected after 2 hours and cells were counted to establish the final yield of freshly purified cells. Alveolar type II cell viability was assessed with trypan blue (Sigma), showing greater than 95% viability. Cell purity was routinely assessed by epithelial cell morphology and immunofluorescence analysis with pan-cytokeratin and pro-surfactant protein C (both positive) as well as α-SMA and CD45 (both negative) of cytocentrifuge preparations of ATII cells. ATII cells used throughout this study demonstrated 95% ± 3% purity. Finally, ATII cells were suspended in Dulbecco's Modified Eagle's Medium (DMEM) plus 10% FCS, 2 mM l-glutamine, 100 U/ml penicillin, and 100 g/ml streptomycin and cultured for 24 hours to allow attachment. Phenotypic characterization was done after this time period. After media change, cells were cultured for a maximum of 3 days in a humidified atmosphere of 5% CO2 at 37°C. The A549 human alveolar type II cell line was purchased from American Type Culture Collection (Rockville, MD, USA) and were cultured in supplemented Roswell Park Memorial Institute (RPMI) 1640 medium at 37ºC in a humidified atmosphere of 5% CO2 in air, as outlined ^[3](#_ENREF_3)^. Cells at 60–70% confluence were serum-deprived by incubation for 12–18 h in RPMI 1640 medium containing 0.1% (v/v) foetal bovine serum prior to stimulation with TGFβ or other agents.

Primary human lung fibroblasts were obtained from lung parenchyma of macroscopically fibrotic affected areas of IPF patients as previously outlined ^[4](#_ENREF_4)^. Lung parenchyma was cut into small pieces, treated with pronase (1 mg/mL; Calbiochem®, Novabiochem®, San Diego, CA, USA) at 37ºC for 30 min, placed in cell culture plates and incubated in DMEM supplemented with 10% foetal calf serum (Sigma, St. Louis, MO, USA), 100 U/mL penicillin/streptomycin and 2% fungizone (GIBCO, Grand Island, NY, USA). After approximately 2 weeks, fibroblasts had grown from the tissue and were passaged by standard trypsinisation. Cells from passages 3–10 were used in all experiments described in the present study. Normal lung fibroblast MRC5 was purchased from American Type Culture Collection (Rockville, MD, USA) and were cultured in 10% FCS supplemented RPMI-1640 medium at 37ºC in a humidified atmosphere of 5% CO2 in air.

For *in vitro* studies, ATII/A549 or primary lung fibroblast/ MRC5 were stimulated with recombinant TGFβ1 (5ng/ml; Sigma Aldrich), or IL-6 (50ng/ml; Sigma Aldrich) with IL-13 (50ng/ml; Sigma Aldrich) combination for the indicated times, replacing culture medium and stimulus every 24 h. TGFβ1 (5ng/ml), IL-6 (50ng/ml) and IL-13 (50ng/ml) have demonstrated to induce cell phenotypic changes such as epithelial to mesenchymal transition (EMT) at the indicated concentrations [^2^](#_ENREF_2)^,^ [^5^](#_ENREF_5)^,^ [^6^](#_ENREF_6).

JSI-124 (selective JAK2/STAT3 inhibitor; 1μM suppress JAK2/STAT3 activation in A549 cells [^7^](#_ENREF_7); Sigma Aldrich), NSC-33994 (selective JAK2 inhibitor; at 1μM completely inhibit JAK2 activity without affecting other tyrosine kinases [^8^](#_ENREF_8); Sigma Aldrich), and 5, 15-DPP (selective STAT3 inhibitor, at 1μM completely inhibit STAT3 activity without affecting other STAT [^9^](#_ENREF_9) ; Sigma Aldrich) were added 30 min before stimulus and remained together with the stimulus until experimental evaluation. None of the drugs affected cell viability assessed with trypan blue (Sigma), showing greater than 95% viability.

**Western blotting analysis**

Western blotting analysis was used to detect changes in human and rat lung tissues, and ATII/A549 and lung fibroblast/MRC5 cell protein expression. Lung tissue or cells were homogenized or scraped from a confluent 25-cm^2^ flask and lysed on ice with a lysis buffer comprising a complete inhibitor cocktail plus 1 mM ethylenediaminetetraacectic acid (Roche Diagnostics Ltd., West Sussex, UK) with 20 mM Tris base, 0.9% NaCl, 0.1% Triton X-100, 1 mM dithiothreitol, and 1 mg/mL pepstatin A. The Bio-Rad assay (Bio-Rad Laboratories Ltd., Herts, UK) was used according to the manufacturer’s instructions to quantify the level of protein in each sample to ensure equal protein loading. Sodium dodecyl sulfate polyacrylamide gel electrophoresis was used to separate the proteins according to their molecular weight. Briefly, 15 µg of proteins (denatured) along with a molecular weight protein marker (Bio-Rad Kaleidoscope marker; Bio-Rad Laboratories) were loaded onto an acrylamide gel consisting of a 5% acrylamide stacking gel stacked on top of a 10% acrylamide resolving gel and run through the gel by application of 100 V for 1 h. Proteins were transferred from the gel to a polyvinylidene difluoride membrane using a wet-blotting method. The membrane was blocked with 5% Marvel in PBS containing 0.1% Tween20 (PBS-T), probed with the following antibodies: rabbit anti-human/rat JAK2 (1:1000) antibody (polyclonal antibody; Novus Biologicals, Abingdon Oxon, UK; catalog no. NBP1-61916), rabbit anti-human/rat phospho(p)-JAK2 (1:1000) antibody (monoclonal antibody; Novus Biologicals, Abingdon Oxon, UK; catalog no. NB110-57144), rabbit anti-human/rat STAT3 (1:1000) antibody (polyclonal antibody; Novus Biologicals, Abingdon Oxon, UK; catalog no. NB100-91973), rabbit anti-human/rat phospho(p)-STAT3 (1:1000) antibody (monoclonal antibody; Novus Biologicals, Abingdon Oxon, UK; catalog no. NB100-80051), mouse anti-human/rat α-SMA (1:1000) antibody (monoclonal antibody Sigma Aldrich, Madrid, Spain; catalog no. A5228), rabbit anti-human/rat collagen type I (1:1000) antibody (polyclonal antibody; Calbiochem Darmstadt, Germany; catalog no. 234167), rabbit anti-rat TGFβ1 (1:1000) antibody (monoclonal antibody; Cell Signaling Technology Inc., Barcelona, Spain; catalog no. 3709S), mouse anti-rat ET-1 (1:1000) antibody (monoclonal antibody; Thermo Scientific, IL, US; catalog no. MA3-005) and rabbit anti-human/rat phospho(p)-Smad3 (1:1000) antibody (monoclonal antibody; Millipore, Madrid, Spain; catalog no. PS1023), goat anti-rat CTGF (1:1000) antibody (polyclonal antibody; Santa Cruz Biotechnology, Madrid, Spain; catalogue no. SC-34772), mouse anti-human E-cadherin (1:1000) antibody (monoclonal antibody; ECM BioScience, Versailles, USA; catalog no. CM1681), rabbit anti-human/rat p21 (1:1000) antibody (polyclonal antibody; Novus Biologicals, Abingdon Oxon, UK; catalog no. NB100-1941), mouse anti-human/rat LC3II (1:1000) antibody (monoclonal antibody; Santa Cruz Biotechnology, Madrid, Spain; catalogue no. SC-376404), rabbit anti-human/rat BCL-2 (1:1000) antibody (polyclonal antibody; Novus Biologicals, Abingdon Oxon, UK; catalog no. NB100-92142), mouse anti-human/rat beclin-1 (1:1000) antibody (monoclonal antibody; Novus Biologicals, Abingdon Oxon, UK; catalog no. NBP1-00084), and normalized to total mouse anti-human/rat β-actin (1:1000) antibody (monoclonal antibody, catalog no. A1978; Sigma). The enhanced chemiluminescence method of protein detection using enhanced chemiluminescence reagents (ECL Plus; Amersham GE Healthcare, Buckinghamshire, UK) was used to detect labeled proteins. Densitometry of films was performed using the Image J 1.42q software (available at http://rsb.info.nih.gov/ij/, USA). Results of target protein expression are expressed as the percentage of the densitometry of the endogenous controls β-actin.

**Real-time RT-PCR and siRNA experiments**

Total RNA was isolated from cells/lung tissue using TriPure^®^ Isolation Reagent (Roche, Indianapolis, USA). The integrity of the extracted RNA was confirmed with Bioanalyzer (Agilent, Palo Alto, CA, USA). Reverse transcription was performed in 300 ng of total RNA with a TaqMan reverse transcription reagents kit (Applied Biosystems, Perkin-Elmer Corporation, CA, USA). cDNA was amplified with specific primers and probes predesigned by Applied Biosystems for JAK2 (Hs01078136_m1), STAT3 (Hs00374280_m1), α-SMA (Hs00559403_m1), α_1_(I)-collagen (collagen type I; Hs00164004_m1) Snail (Hs00195591_m1), Slug (Hs00161904_m1), E-cadherin (Hs01023894_m1), β-actin (Hs01060665) for human tissue, and collagen type I (Rn00301649_m1), CTGF (Rn00573960_g1), ET-1 (Rn00561129_m1), TGFβ1 (Rn00572010_m1) and β-actin (Rn00667869) for rat tissue in a 7900HT Fast Real-Time PCR System (Applied Biosystems) using Universal Master Mix (Applied Biosystems).

Expression of the target gene was expressed as the fold increase or decrease relative to the expression of GAPDH as an endogenous control. The mean value of the replicates for each sample was calculated and expressed as the cycle threshold (Ct). The level of gene expression was then calculated as the difference (ΔCt) between the Ct value of the target gene and the Ct value of GAPDH. The fold changes in the target gene mRNA levels were designated 2^-ΔCt^.

Small interfering RNA (siRNA), including the scrambled siRNA control (identification no. 4390843), was purchased from Ambion (Huntingdon, Cambridge, UK). JAK2 (identification no. s7250) and STAT3 (identification no. s743) gene-targeted siRNA were designed by Ambion. A549 and MRC5 cells were transfected with siRNA (50 nM) in serum and antibiotic-free medium. After 6 h, the medium was aspirated and replaced with medium containing serum for a further 42 h before cell stimulation. The transfection reagent used was lipofectamine-2000 (Invitrogen, Paisley, UK) at a final concentration of 2 µg/mL. JAK2 and STAT3 knockdown were evaluated in A549 and MRC5 cells by western blot and RT-PCR using JAK2/STAT3 antibodies and JAK2/STAT3 primers and probe described above. JAK2/STAT3 expression in JAK2/STAT3 knockdown experiments was always lesser than the 10% of JAK2/STAT3 expression in siRNA(-) control cells (data not shown).

**Histological and Immunohistochemical Studies**

Lung histology was conducted as previously reported ^[10](#_ENREF_10)^. Tissue blocks (4 μm thickness) were stained with haematoxylin-eosin for assessment of the fibrotic injury and pulmonary artery remodeling, and with Masson’s trichrome (Sigma-Aldrich, Madrid, Spain) to detect collagen deposition. Severity of lung fibrosis was scored on a scale from 0 (normal lung) to 8 (total fibrotic obliteration of fields) according to Ashcroft [^11^](#_ENREF_11).

For immunohistochemical analysis of rat and human lungs, tissue was fixed and embedded in paraffin, cut into sections (4-6 µm) and incubated with JAK2, pJAK2, STAT3, pSTAT3, collagen type I, LC3II, beclin-I, Bcl-2 and p21 antibodies for 24 h at 4°C. A secondary anti-rabbit goat or anti-mouse antibody (1:100; Vector Laboratories, Burlingame, CA) with avidin-biotin complex/horseradish peroxidase was used for immunohistochemistry. The non-immune IgG isotype control was used as negative control and gave negative for all samples.

**ELISA**

IL-6 and IL-13 cytokines were analysed in cell culture supernatants of human ATII and fibroblast using using commercially available ELISA quantikine® ELISA human IL-6 (R&D systems, Madrid, Spain; catalog no. D6050) and IL-13 (R&D systems, Madrid, Spain; catalog no. D1300B) kits, and in bronchoalveolar lavage (BAL) fluid of rats using the ELISA rat IL-6 (Invitrogen™, Madrid, Spain, catalog no. KRC0061) and IL-13 (Invitrogen™, Madrid, Spain, catalog no. KRC0132) kits according to the manufacturer’s protocol.

**Wound repair**

Wounding repair studies were carried out in IPF primary human lung fibroblasts. Fibroblasts were cultured in 6-well plates as described in methods and pre-treated with pharmacologic modulators for 30 minutes. A scrape-wounded was done using a sterile p200 pipette tip by one perpendicular linear scratch, creating a wound of ̴ 1mm width across the diameter of the 6-well plates. Cells were then washed two times to eliminate floating and dead cells. After washing, cell culture media was added with or without pharmacologic modulators and IL-6/IL-13 stimulus. To be sure that the wound area measures were done in the same place during wound closure, a black line was painted to the bottom of each plate and wound areas were measured in the intersection of the wound and the black line. Wound closure was monitored immediately after initial wounding using a 5x phase contrast objective lens and was digitally captured at regular time intervals after wounding until repair was complete. Wound areas were analysed using Image J 1.42q software (available at; http://rsb.info.nih.gov/ij/, USA); the extent of repair was calculated and expressed as a percentage of the original wound area.

**Proliferation assay**

IPF primary human lung fibroblast proliferation was measured by colorimetric immunoassay based on BrdU incorporation during DNA synthesis using a cell proliferation enzyme-linked immunosorbent assay BrdU kit (Roche, Mannheim, Germany) according to the manufacturer’s protocol as previously outlined [^12^](#_ENREF_12). Semiconfluent cultured fibroblasts were grown in 96-well plates at a density of 3x10^3^ cells/well and incubated for 24 h. Cells were then treated with different JAK2/STAT3 inhibitors during 30 min and stimulated with culture medium with FBS1% or FBS 10% as indicated for 48 h. The 450 nm absorbance was quantified using a microplate spectrophotometer (Victor 1420 Multilabel Counter, PerkinElmer). Proliferation data refer to the absorbance values of BrdU-labeled cellular DNA content per well. Stimulation is expressed as absorbance units.

**Intratracheal bleomycin animal model**

Experimentation and handling were performance in accordance with the guidelines of the Committee of Animal Ethics and Well-being of the University of Valencia (Valencia, Spain). Rat studies used pathogen-free male wistar rats (Harlan Iberica^®^, Barcelona, Spain) at 12 weeks of age which are reported to mount a robust early inflammatory response (days 1-7 of procedure) followed by fibrotic lung remodeling secondary to bleomycin (days 14-28) [^13^](#_ENREF_13). Rats were housed with free access to water and food under standard conditions: relative humidity 55 ± 10 %; temperature 22 ± 3ºC; 15 air cycles/ per hour; 12/12 h Light/Dark cycle. Rats were anaesthetized with ketamine/medetomidine and then a single dose of bleomycin at 3.75 U/kg (dissolved in 200 µL of saline) was administered intratracheally via the endotracheal route.[^14^](#_ENREF_14) This dose of bleomycin reproducibly generated pulmonary fibrosis in previous experiments.[^15^](#_ENREF_15) Sham treated rats received the identical volume of intratracheal saline instead of bleomycin. This procedure fixed experimentation day 1. Dose of JSI-124 was selected based in agreement with previous *in vivo* animal studies (1mg/kg/day, i.p) [^16^](#_ENREF_16) and was administered from day 14 to day 28 of procedure as therapeutic protocol.

JSI-124 was prepared immediately prior to use. JSI-124 was prepared as DMSO solution. The control group received DMSO as vehicle. The account for experimental groups was estimated in a number of 10 rats (n=10): (i) saline serum + pharmaceutical vehicle; (ii) saline serum + JSI-124 (1 mg/Kg/day); (iii) bleomycin + pharmaceutical vehicle; (iv) JSI-124 (1 mg/Kg/day). With these doses of JSI-124, no adverse effects were observed during the experiments. Results obtained for the group of saline serum + JSI-124 were identical to those of saline group. Therefore we did not include because space restrictions in main manuscript. At the end of the treatment period (day 28), rats were sacrificed by a lethal injection of sodium pentobarbital followed by exsanguination. After opening the thoracic cavity, trachea, lungs and heart were removed *en bloc*. BAL was performed (see below) and lungs were weighed and then processed for histological, biochemical or molecular biology studies.

**Bronchoalveolar lavage**

At the end of experiments BAL fluid was recovered following five consecutive washes of the right lung with 0.6 mL aliquots of saline flushed through a tracheal cannula. Cell suspensions were concentrated by low speed centrifugation (150g, 5 min) and cells resuspended in phosphate buffer. Total cell counts were made in a haemocytometer. Differential cell counts were determined from cytospin preparations by counting about 300 cells stained with May-Gruenwald-Giemsa. Total protein content in BAL fluid supernatants was measured by using the BCA Protein Assay Kit (Pierce Chemical Co, Rockford IL, USA) according to the manufacturer’s instructions. Results are expressed in µg protein per mL. BAL fluid supernatants were stored at -80°C for measurements of IL-6 and IL-13.

**Micro-CT analysis**

Rats were anesthesized with intraperitoneal mixture of ketamin (70 mg/Kg) and medetomidin (0.25 mg/kg). The animals were introduced into the micro- computer tomography (CT)-PET-SPECT (Albira, Oncovision™, Valencia, Spain) in supine position in a cradle made of plexiglas, and capture of micro-CT images were acquired at day 0, 7, 14 and 28. The time of each capture was 30 min (until 300000 counts). Lung images were taken as follow: planar scintigraphy in anterior (A), posterior (P) left and right lateral (LL and RL), and left and right posterior oblique (LPO and RPO) views.

X-ray captures were acquired through Step&Shoot modality using 400μA of current intensity, 45 kV of voltage and 0.5mm attenuation filter.

The images were reconstructed with filtered back projection algorithm using the Albira Suite 5.0 software (OncoVision, Valencia, Spain). The combination of acquisition and reconstruction result in a final whole image matrix of 255*255*255 mm with a pixel size of 0.25*0.25*0.25 mm. 252 image sections corresponding to the whole lung images were acquired in each capture and analyzed with the PMOD™ software selecting a whole 3D volume of interest (VOI). Densitometric analysis of the extension of fibrosis was quantified using micro-CT images as Houndsfield Units (HU). The mean of HU units per pixel was calculated.

**Statistical analysis**

Statistical analysis of results was carried out by parametric (animal and cellular studies) or non-parametric (human tissue studies) analysis as appropriate. *P* < 0.05 was considered statistically significant. Non-parametric tests were used to compare results from human samples of control patients and IPF patients. In this case, data were displayed as medians and interquartile range values. When the comparisons concerned only 2 groups, between-group differences were analyzed by the Mann Whitney test. Results from animal and cellular *in vitro* mechanistic cell experiments were expressed as mean ± SE of n experiments since normal distribution for each data set was confirmed by histogram analyses and Kolmogorov–Smirnov test. In this case, statistical analysis was carried out by parametric analysis. Two-group comparisons were analysed using the two-tailed Student’s paired t-test for dependent samples, or unpaired t-test for independent samples. Multiple comparisons were analysed by one-way or two-way analysis of variance followed by Bonferroni post hoc test.

**Supplemetary Figures**


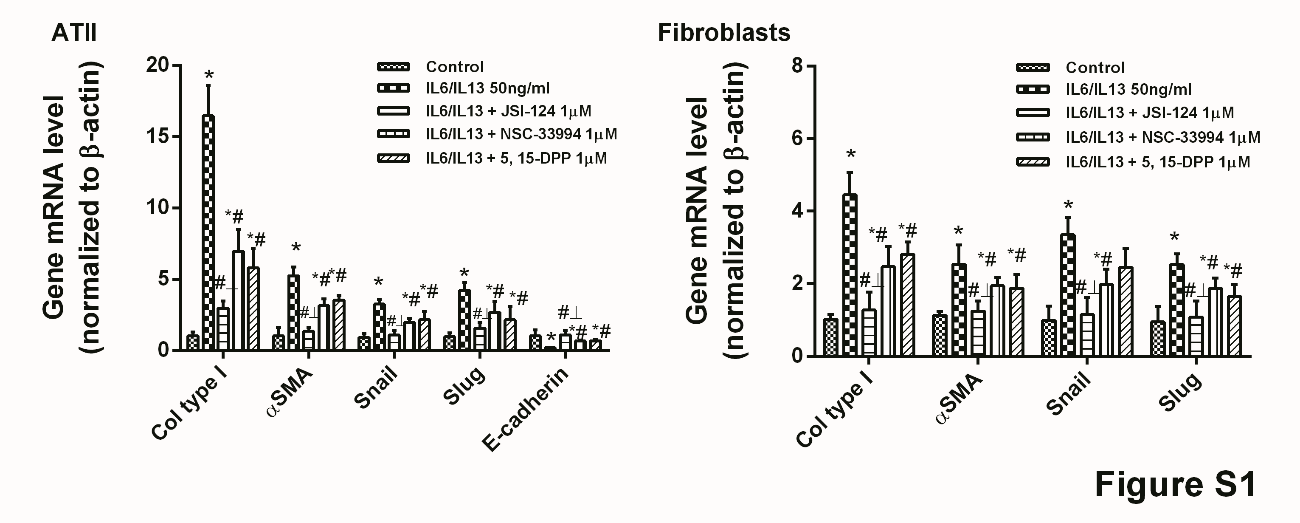


**Supplementary Figure S1**

Primary ATII and lung fibroblasts were isolated from lungs of IPF patients and preincubated with 5,15 DPP 1µM (p-STAT3 inhibitor), NSC33994 1µM (p-JAK2 inhibitor) or JSI-124 1µM (dual p-JAK2/p-STAT3 inhibitor) for 30 min and stimulated with IL-6/IL-13 during 72h. Total RNA was extracted to analyze the mRNA expression of mesenchymal markers alpha smooth muscle actin (αSMA), collagen type I, Snail, Slug and epithelial marker E-cadherin. Data are expressed as ratios to β-actin mRNA levels. Results are expressed as means (SE) of *n* = 4 (cells from four IPF patients) experiments per condition. Two-way ANOVA followed by post hoc Bonferroni tests. *p < 0.05 related to solvent controls; #p < 0.05 related to IL-6/IL-13; ┴ < 0.05 related 5,15 DPP or NSC33994.


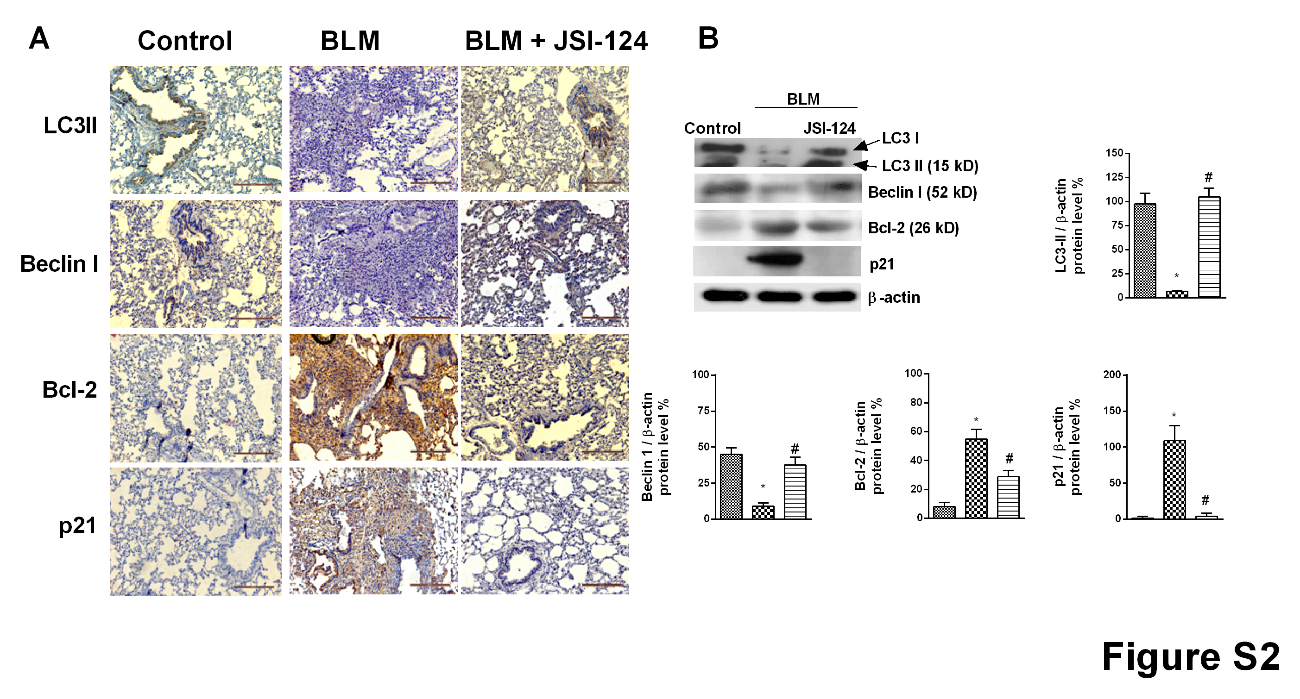


**Figure S2. JSI-124 reduces senescence and anti-apoptotic pathways and improves autophagy in a model of bleomycin-induced lung fibrosis.**

Wistar rats received a single intratracheal dose of bleomycin (BLM; 3.75 U/kg) on day 1. JSI-124 (1mg/kg/day i.p) or vehicle was administered from day 14 until analysis at day 28 (*n* = 10 per group). Immunohistochemistry for senescence p21, anti-apoptotic BCL-2 and autophagy LC3I/II and beclin I markers were analysed by (*A*) immunohistochemistry and (*B*) western blot. Data are expressed as ratios to β-actin protein levels. Representative images of immunohistochemistry and western blot images are shown (scale bar: 100µm). Results are expressed as means ± SE, *n* = 10. Statistical significance was assessed using one-way ANOVA followed by a Bonferroni *post hoc* test. **P* < 0.05 *vs.* control, #*P* < 0.05 *vs.* BLM.

**References**

1. Raghu G, Rochwerg B, Zhang Y, Garcia CA, Azuma A, Behr J, et al. An Official ATS/ERS/JRS/ALAT Clinical Practice Guideline: Treatment of Idiopathic Pulmonary Fibrosis. An Update of the 2011 Clinical Practice Guideline. Am J Respir Crit Care Med 2015; 192:e3-19.

2. Milara J, Navarro R, Juan G, Peiro T, Serrano A, Ramon M, et al. Sphingosine-1-phosphate is increased in patients with idiopathic pulmonary fibrosis and mediates epithelial to mesenchymal transition. Thorax 2012; 67:147-56.

3. Mata M, Sarria B, Buenestado A, Cortijo J, Cerda M, Morcillo EJ. Phosphodiesterase 4 inhibition decreases MUC5AC expression induced by epidermal growth factor in human airway epithelial cells. Thorax 2005; 60:144-52.

4. Milara J, Serrano A, Peiro T, Gavalda A, Miralpeix M, Morcillo EJ, et al. Aclidinium inhibits human lung fibroblast to myofibroblast transition. Thorax 2012; 67:229-37.

5. Cao H, Zhang J, Liu H, Wan L, Zhang H, Huang Q, et al. IL-13/STAT6 signaling plays a critical role in the epithelial-mesenchymal transition of colorectal cancer cells. Oncotarget 2016; 7:61183-98.

6. Xie G, Yao Q, Liu Y, Du S, Liu A, Guo Z, et al. IL-6-induced epithelial-mesenchymal transition promotes the generation of breast cancer stem-like cells analogous to mammosphere cultures. Int J Oncol 2012; 40:1171-9.

7. Blaskovich MA, Sun J, Cantor A, Turkson J, Jove R, Sebti SM. Discovery of JSI-124 (cucurbitacin I), a selective Janus kinase/signal transducer and activator of transcription 3 signaling pathway inhibitor with potent antitumor activity against human and murine cancer cells in mice. Cancer Res 2003; 63:1270-9.

8. Kiss R, Polgar T, Kirabo A, Sayyah J, Figueroa NC, List AF, et al. Identification of a novel inhibitor of JAK2 tyrosine kinase by structure-based virtual screening. Bioorg Med Chem Lett 2009; 19:3598-601.

9. Uehara Y, Mochizuki M, Matsuno K, Haino T, Asai A. Novel high-throughput screening system for identifying STAT3-SH2 antagonists. Biochem Biophys Res Commun 2009; 380:627-31.

10. Almudever P, Milara J, De Diego A, Serrano-Mollar A, Xaubet A, Perez-Vizcaino F, et al. Role of tetrahydrobiopterin in pulmonary vascular remodelling associated with pulmonary fibrosis. Thorax 2013; 68:938-48.

11. Ashcroft T, Simpson JM, Timbrell V. Simple method of estimating severity of pulmonary fibrosis on a numerical scale. J Clin Pathol 1988; 41:467-70.

12. Cortijo J, Milara J, Mata M, Donet E, Gavara N, Peel SE, et al. Nickel induces intracellular calcium mobilization and pathophysiological responses in human cultured airway epithelial cells. Chem Biol Interact 2009.

13. Hemnes AR, Zaiman A, Champion HC. PDE5A inhibition attenuates bleomycin-induced pulmonary fibrosis and pulmonary hypertension through inhibition of ROS generation and RhoA/Rho kinase activation. Am J Physiol Lung Cell Mol Physiol 2008; 294:L24-33.

14. Bivas-Benita M, Zwier R, Junginger HE, Borchard G. Non-invasive pulmonary aerosol delivery in mice by the endotracheal route. Eur J Pharm Biopharm 2005; 61:214-8.

15. Mata M, Ruiz A, Cerda M, Martinez-Losa M, Cortijo J, Santangelo F, et al. Oral N-acetylcysteine reduces bleomycin-induced lung damage and mucin Muc5ac expression in rats. Eur Respir J 2003; 22:900-5.

16. Nefedova Y, Nagaraj S, Rosenbauer A, Muro-Cacho C, Sebti SM, Gabrilovich DI. Regulation of dendritic cell differentiation and antitumor immune response in cancer by pharmacologic-selective inhibition of the janus-activated kinase 2/signal transducers and activators of transcription 3 pathway. Cancer Res 2005; 65:9525-35.
